# Supplementary material for: The interactome of intact mitochondria by cross-linking mass spectrometry provides evidence for coexisting respiratory supercomplexes
Source: Mol Cell Proteomics. 2017 Dec 8;17(2):216–32. doi: 10.1074/mcp.RA117.000470 (PMC5795388; doi:10.1074/mcp.RA117.000470)
Supplement: Supplemental Data [file supp_RA117.000470_133922_0_supp_23978_fzffwf.zip › spectra_annotation/mito_DR_spectra_annotation/384-1-6-1-5-1.pdf]

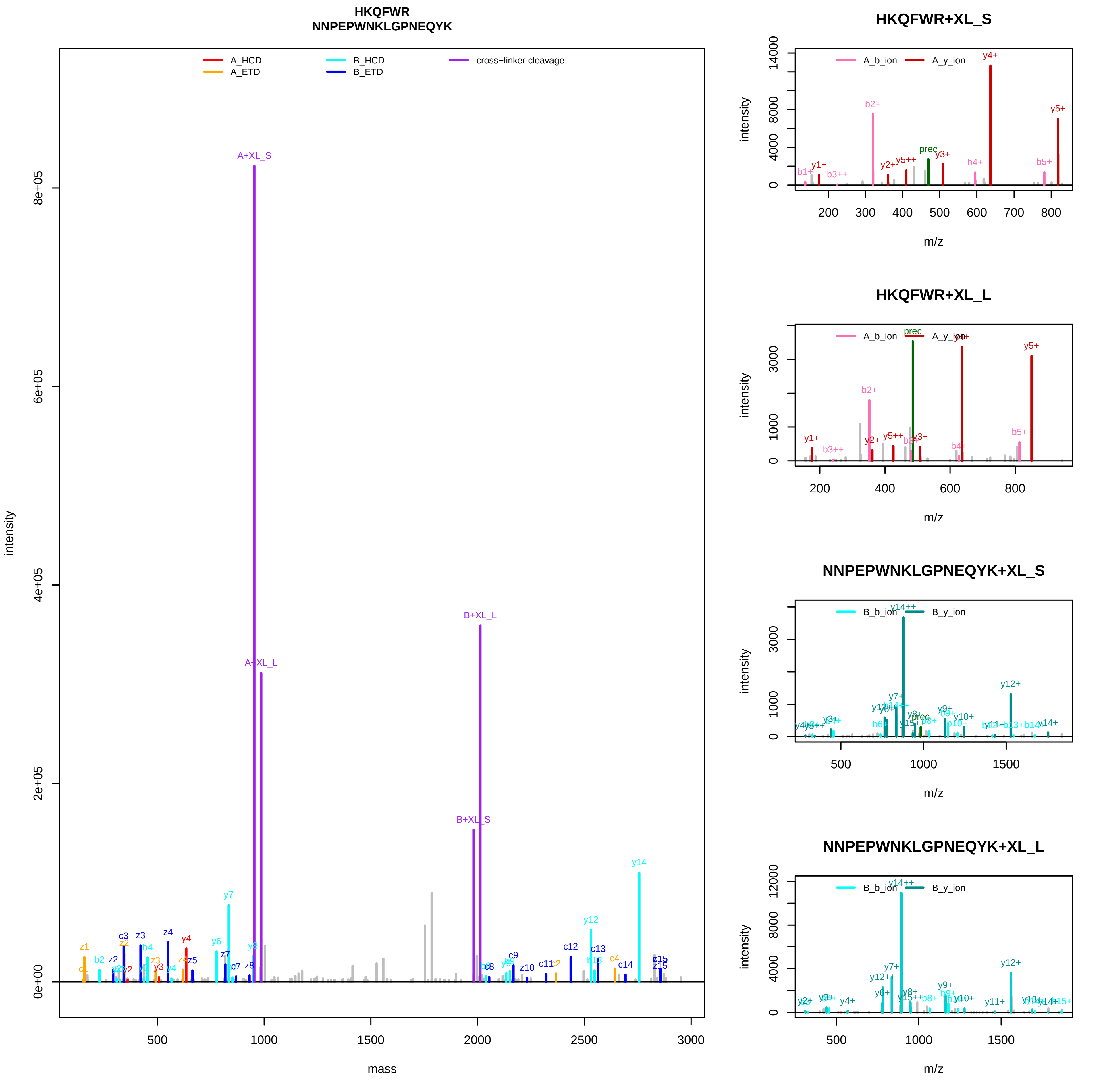
HKQFWR
NNPEPWNKLGPNEQYK
A_HCD
A_ETD
B_HCD
B_ETD
cross−linker cleavage
A+XL_S
A+XL_L
B+XL_L
B+XL_S
intensity
mass
0e+00
2e+05
4e+05
6e+05
8e+05
500
1000
1500
2000
2500
3000
z1
c1
b2
z2
c3
z2
y2
z3
b4
z4
y4
y3
z3
y4
z4
z5
y6
y7
z7
c7
z8
y8
c8
y9
c9
z10
c11
c2
c12
y12
c13
b13
c4
c14
y14
z15
z15
HKQFWR+XL_S
A_b_ion
A_y_ion
b1+
y1+
b3++
b2+
y2+
y5++
prec
y3+
b4+
y4+
b5+
y5+
HKQFWR+XL_L
NNPEPWNKLGPNEQYK+XL_S
B_b_ion
B_y_ion
y14++
y12+
y7+
y9+
y10+
y14+
NNPEPWNKLGPNEQYK+XL_L
y12++
y8+
y11+
y13+
m/z
0
4000
8000
14000
12000
1000
3000
200
300
400
500
600
700
800
1500
